# Autism and chronic ill health: an observational study of symptoms and diagnoses of central sensitivity syndromes in autistic adults

**DOI:** 10.1186/s13229-022-00486-6

**Published:** 2022-02-14

**Authors:** Sarah Grant, Sam Norton, Ricarda F. Weiland, Anke M. Scheeren, Sander Begeer, Rosa A. Hoekstra

**Affiliations:** 1 Department of Psychology, Institute of Psychiatry, Psychology and Neuroscience, King’s College London, London, SE5 8AF UK; 2 Faculty of Behavioural and Movement Sciences, Clinical Developmental Psychology, Vrije Universiteit Amsterdam, Van der Boechorststraat 7, 1081 BT Amsterdam, Netherlands

**Keywords:** Autism, Central sensitisation, Sensory processing, Sensory sensitivity, Fibromyalgia, Fatigue, Chronic pain

## Abstract

**Background:**

Autistic adults, particularly women, are more likely to experience chronic ill health than the general population. Central sensitivity syndromes (CSS) are a group of related conditions that are thought to include an underlying sensitisation of the central nervous system; heightened sensory sensitivity is a common feature. Anecdotal evidence suggests autistic adults may be more prone to developing a CSS. This study aimed to investigate the occurrence of CSS diagnoses and symptoms in autistic adults, and to explore whether CSS symptoms were related to autistic traits, mental health, sensory sensitivity, or gender.

**Methods:**

The full sample of participants included 973 autistic adults (410 men, 563 women, mean age = 44.6) registered at the Netherlands Autism Register, who completed questionnaires assessing autistic traits, sensory sensitivity, CSS, physical and mental health symptoms. The reliability and validity of the Central Sensitization Inventory (CSI) in an autistic sample was established using exploratory and confirmatory factor analyses. Chi^2^ analyses, independent *t*-tests, hierarchical regression and path analysis were used to analyse relationships between CSS symptoms, autistic traits, measures of mental health and wellbeing, sensory sensitivity, age and gender.

**Results:**

21% of participants reported one or more CSS diagnosis, and 60% scored at or above the clinical cut-off for a CSS. Autistic women were more likely to report a CSS diagnosis and experienced more CSS symptoms than men. Sensory sensitivity, anxiety, age and gender were significant predictors of CSS symptoms, with sensory sensitivity and anxiety fully mediating the relationship between autistic traits and CSS symptoms.

**Limitations:**

Although this study included a large sample of autistic adults, we did not have a control group or a CSS only group. We also could not include a non-binary group due to lack of statistical power.

**Conclusions:**

CSS diagnoses and symptoms appear to be very common in the autistic population. Increased awareness of an association between autism and central sensitisation should inform clinicians and guide diagnostic practice, particularly for women where CSS are common and autism under recognised.

**Supplementary Information:**

The online version contains supplementary material available at 10.1186/s13229-022-00486-6.

## Background

Autistic people are more likely to experience a broad range of physical health issues [1], including chronic disease and premature mortality [2, 3] and have poorer general health outcomes [4] than the wider population. However, the underlying mechanisms are not yet well established. One group of physical problems colloquially thought to be more prevalent in autistic people are ‘central sensitivity syndromes’ (CSS) including myalgic encephalomyelitis/chronic fatigue syndrome (ME/CFS), fibromyalgia syndrome (FMS), migraine, irritable bowel syndrome (IBS) restless legs syndrome (RLS) and temporomandibular joint disorder (TMJD). CSS are thought to have central sensitisation, or augmented sensory signalling of the central nervous system, as a core component [5]; symptoms include fatigue, chronic pain and sensory hypersensitivity. In the general population, prevalence estimates of CSS vary 0.2–20% [6–12] depending on type of syndrome and country. For example, fibromyalgia is thought to be prevalent at between 0.2 and 6.6% in the general population [6], but the global prevalence for IBS has been more difficult to ascertain, with ranges varying from 5.8% in the Middle East and Africa, up to 17.5% in Latin America [10].

A core feature common to both autism [13] and CSS [14] is sensory sensitivity. While sensory research in autism has been more focussed on altered experience and heightened sensory sensitivity across all modalities [15], CSS research has been centred around pain [16]. Therefore, whilst CSS studies have acknowledged that general sensory sensitivity, and not just pain, is part of central sensitisation [17–21], the mechanisms of individual differences in sensory sensitivity within this population, as well as the neurodivergent and general population, are still unclear [22]. Studies on sensory differences in autism are plentiful [13, 15, 23, 24] but research specifically on the autistic pain experience is more limited. Research on acute pain in autism, and quantitative sensory testing studies, has suggested that autistic people have a normal or hypersensitive physiological response to acute pain, but may express pain differently [25–28] and also experience more pain-related anxiety [29], but it has not yet been established whether this anxiety contributes to, or is caused by, altered pain sensitivity. Neural differences in the sustained pain response have also been found in autistic people [30] but to date there have been no studies exploring the phenomenon of central sensitisation in the autistic population.

Sensory sensitivity is not the only commonality between autism and CSS. The autistic and CSS communities both experience psychosocial factors that can reduce their physical and mental wellbeing, including (but not limited to) poor mental health [31–33], trauma [34, 35], stigma and discrimination [36–39], socioeconomic disparity [40, 41] and poor access to or experiences with healthcare [42, 43]. CSS have a troubled history in the research literature, with many clinicians still referring to them as somatoform disorders despite considerable evidence to the contrary [44, 45]. Psychosocial factors have been shown to play a complex role in the development and maintenance of CSS and chronic pain [46] however how these factors affect physical health in the autistic community is under-explored.

Research looking directly at an association between autism and CSS is limited. Paediatric studies have highlighted a higher incidence of neurodevelopmental disorders in children with chronic pain [47, 48] and/or CSS [49, 50], but there is little equivalent research in adults. There is, however, growing awareness of a link between autism and genetic connective tissue disorders, particularly joint hypermobility- related disorders [51, 52] and the Ehlers-Danlos syndromes [53]. These conditions often co-occur with CSS [54–56], but more research is needed to determine whether this directly translates to an association between autism and CSS.

CSS are much more commonly diagnosed in women than in men [57]. Women are also proposed to have greater pain sensitivity [58, 59] and heightened central sensitisation [60] although how much of this difference is truly gender specific [61] and how much can be attributed to gender bias [62, 63] is unclear. Gender is also an important predictor of an autism diagnosis and physical health in autism. Autism has historically been under recognised [64] and diagnosed later [65] in women, and autistic women appear to experience a greater range of co-occurring physical conditions than autistic men [66]. Whether CSS are more common in autistic women has not been explored.

Our study aimed to investigate the rates of CSS and CSS symptoms in a sample of autistic adults. We first examined the dimensionality and reliability of the Central Sensitization Inventory (CSI), a widely used CSS measure [67], in this autistic sample. We hypothesized that, given the link between autism and sensory sensitivity, and sensory sensitivity and central sensitisation, as well as the high incidence of co-occurring conditions in autism, CSS symptoms would be common in autistic adults and may be more prevalent than observed in the general population. We also hypothesized that, since high scores on measures of autistic traits correlate with autism diagnoses [68], then autistic traits would be positively associated with CSS symptoms as measured through the CSI.

We also postulated that higher CSI scores would be associated with greater sensory sensitivity, higher anxiety and depression scores and poorer physical health and subjective well-being, and we predicted that autistic women would report greater sensory sensitivity and more CSS symptoms than autistic men. Lastly, as sensory sensitivity, anxiety and chronic pain have been linked together in previous studies [29, 69] we considered whether sensory sensitivity or anxiety might mediate a relationship between autism and CSS.

## Methods

### Participants

The sample comprised 973 adults (410 men, 563 women) all of whom had been formally diagnosed with autism. Nine people who indicated their gender was “other” were excluded due to the small sample size.

The mean age of the sample was 44.6 years (SD = 13.58), with men (Mean = 48.7, SD = 13.42) significantly older than women (Mean = 41.7, SD = 12.93, *p* < 0.001). One participant had not confirmed their age. 18.6% of the sample had completed a university degree, 21.5% a higher professional education, 16.3% a vocational education, 20.6% had another type of education and 23.0% had not specified their level of education. Participants were asked about ethnicity, and the vast majority indicated Dutch heritage, with only 2% indicating non-Western heritage either through their own or their parents’ country of origin.

Participants were recruited through the Netherlands Autism Register (NAR www.nederland- sautismeregister.nl/english/), a longitudinal autism research volunteer register that is administered on an annual basis to autistic people and/or their legal representatives. The data collection for this study was self-report and was part of an ongoing wave of NAR surveys. Due to the nature of the NAR, some questionnaires in this study have a greater number of participants than others; for example, the SPQ was completed in 2016 when the NAR had fewer participants than in 2019 when the CSI was completed. For this study, participants were asked questions as part of the overall survey rather than being specifically recruited, ensuring we minimised bias in the recruitment process.

### Measures

#### Central sensitisation

The Central Sensitization Inventory [70] was developed as a valid and reliable self-report measure for symptoms of central sensitisation, and later posited as a possible screening instrument. Part A of the CSI questionnaire comprises items relating to symptoms of CSS as identified in a literature search by the original developers of the CSI. Each item is measured on a five-point Likert scale ranging from 0 ‘never’ to 4 ‘always’ (for item content please see Table 1). A cut-off score of 40 on Part A was determined to best distinguish between CSS and non-CSS patients on the original CSI scale [67]. This study used the Dutch translation of the CSI [71] which also uses a cut-off of 40 and has been shown to discriminate well between chronic pain patients and healthy controls, with good internal consistency and test–retest reliability. All 973 participants included in the present study completed the CSI part A in full.

#### CSS diagnoses

Part B of the original CSI contains a list of CSS diagnoses and related disorders. Several health conditions from Part B—fibromyalgia syndrome (FMS), myalgic encephalomyelitis/chronic fatigue syndrome (ME/CFS), irritable bowel syndrome (IBS), restless legs syndrome (RLS), and temporomandibular joint disorder (TMJD)—were included in the 2019 wave of the NAR data collection, with participants able to select them as co-occurring conditions if relevant. The NAR also includes reference to migraine, but within a field entitled “headache/migraine”. It was felt that this category was too broad to accurately identify those participants with migraines versus other types of headache, and therefore this condition was excluded from the analysis. Seven hundred and thirty-three participants completed the section on co-occurring conditions. Participants who indicated one or more of the five included CSS diagnoses were flagged as “diagnosed CSS” with all others, including those who did not answer the questions, flagged as not diagnosed.

#### Physical health

Participants were asked to rate their physical health from 0 to 10 with 0 being the poorest health and 10 being good physical health. This question is asked in each wave of the Netherlands Autism Register. All 973 participants had completed the physical health scale.

#### Sensory sensitivity

The 35 item Sensory Perception Quotient (SPQ) was developed to assess sensory sensitivity in adults with and without autism, and shows good internal consistency and validity [15, 24, 72]. It is assessed on a four-point Likert scale across five sensory modalities. Items range from 0 ‘strongly agree’ to 3 ‘strongly disagree’. Lower scores on the SPQ indicate higher sensory sensitivity, and higher scores lower sensitivity. SPQ data was collected in the 2016 wave of the Netherlands Autism Register and we therefore had data from fewer participants for this questionnaire (*n* = 393). Cronbach’s *α* = 0.880 for the SPQ in this sample.

#### Autistic traits

Autistic traits were measured using the 28 item AQ-Short [73], an abridged version of the 50 item Autism Spectrum Quotient or AQ [74]. Items are scored on a four-point Likert scale ranging from 1 ‘definitely agree’ to 4 ‘definitely disagree’. 13 of the 28 items are reverse scored where ‘agree’ responses are characteristic for autism. This measure has been evaluated in Dutch and English samples and was found to have good reliability, sensitivity and specificity [73, 75]. The AQ is administered when participants register with the NAR, and therefore although the year completed varied per person, all 973 participants had completed this measure.

#### Anxiety and depression

The Hospital Anxiety and Depression Scale or ‘HADS’ [76], consists of two subscales and is used to identify anxiety (HADS-A) and depression (HADS-D) in non-psychiatric patients. Each subscale contains seven items ranging from 0 to 3, with 3 indicating greater symptom severity. The dimensional structure of the HADS has been shown to be stable across groups in Dutch samples, with good sensitivity and specificity [77]. HADS data was collected in the 2018 wave of the Netherlands Autism Register and 636 participants had completed the questionnaire in full. Cronbach’s *α* = 0.864 for the HADS in this sample.

#### Subjective wellbeing

Subjective wellbeing was assessed using a composite score from three separate measures, the Subjective Happiness Scale [78], the Satisfaction with Life Scale [79] and the Cantril ladder [80]. Previous psychometric research has shown that combining these measures in a dimensional score of overall wellbeing (range 2–73) is reliable and valid [81]. The Subjective Happiness Scale has four items on a Likert scale from 1 ‘strongly disagree’ to 7 ‘strongly agree’, with higher scores indicating greater happiness. This measure was completed in the 2016 wave of the NAR. The Satisfaction with Life Scale uses the same Likert scale but with five items related to life satisfaction. The Cantril ladder uses an 11-point scale to evaluate general quality of life, with 0 indicating the worst possible life and 10 the best, and this is completed every year of the NAR. In total, 418 participants had completed all three measures and had a subjective wellbeing score.

### Statistical analyses

The CSI has not previously been validated in the autistic population. To establish the factor structure of the Dutch CSI in this sample of autistic adults, an exploratory factor analysis (EFA) was conducted on a random split half of the sample using a promax rotation and weighted least squares extraction method, with confirmatory factor analyses (CFA) using WLSMV estimator performed on the remaining half of the sample, in which we compared the factor structure indicated by the EFA, and the factor structures reported in previous English CSI [70] and Dutch CSI [71] studies in non-autistic samples as well as a bi-factor structure, as proposed by Cuesta-Vargas et al. [82], whereby the covariance between CSI items was accounted for through one general factor and four orthogonal factors. Model fit was interpreted using the Root Mean Square Error of Approximation (RMSEA), Comparative Fit Indicator (CFI), and Tucker-Lewis indicator (TLI). Optimal fit is indicated by values of 0 for the RMSEA and 1 for the CFI and TLI. Criteria for an ‘acceptable fit’ was an RMSEA < 0.1, CFI/TFI > 0.9. Criteria for an ‘excellent fit’ were RMSEA < 0.06, CFI/TLI > 0.95 < 1.0[83]. EFA and CFA analyses were conducted using MPlus version 8.2 [84] and a reliability analysis of the CSI was conducted in SPSS 25.0 [85]. The factor structure of the CSI in men and women was further investigated through multi-group exploratory and confirmatory factor analyses to confirm measurement equivalence; specifically, testing configural, metric and scalar equivalence. Configural equivalence was assumed where the same factor structure was identified as optimal in EFA and CFA. Where this holds it implies that the scale captures the same construct in both groups. Metric and scalar equivalence were tested using multi-group CFA sequentially confirming that factor loadings (metric) and thresholds (scalar) for each item were equivalent. If configural but not metric or scalar equivalence holds then scale scores are not comparable across groups.

Two additional variables were created, one to indicate whether a participant scored above or below the clinical cut-off of 40 on Part A of the CSI (high or low CSI) and the second to indicate whether the participant reported a CSS of FMS, CFS, RLS, IBS and/or TMJD in the physical complaints section. Data were tested for normality and homogeneity of variance. We used independent samples *t* tests and Chi^2^ tests to analyse CSS group differences (high versus low CSI, and those with and without a CSS diagnosis), and gender group differences. Where relevant, analyses were corrected for multiple testing using Bonferroni correction. Exploratory analyses were then performed. A four-stage hierarchical regression analysis was used to explore the hypothesis that autistic traits, sensory sensitivity and anxiety might significantly predict CSS symptoms, with age and gender included in stage one as controls, and each construct added in a separate stage to explore their effect on the variance in CSI scores. Path analyses were conducted to investigate whether sensory sensitivity or anxiety might mediate the relationship between autistic traits and CSS symptoms.

## Results

### Descriptive statistics

Out of all 973 participants, 208 (21.4%) had a formally diagnosed CSS from the list of included conditions; 41 participants indicated having more than one CSS (see Additional file 1: Table S1 for full list). Eighty-seven percent of participants with a disclosed CSS diagnosis scored at or above the clinical cut-off of 40 on the CSI; participants with a disclosed CSS scored significantly higher on the CSI than those without, *t* (971) =  − 13.214, *p* < 0.001, 95% CI [− 17.083 − 12.665], *d* =  − 1.033. 582 participants (59.8% of the sample) scored at or above the clinical cut-off of 40 on the CSI but only 31% of the ‘high CSI’ group had a formal CSS diagnosis.

### Validation and reliability of the central sensitisation inventory (CSI)

We analysed the psychometric properties of the CSI since it had not been used before in an autistic sample. Exploratory factor analysis in a randomly selected half of the sample indicated a five-factor solution provided the best explanation of the CSI inter-item covariances (RMSEA 0.051, SRMR 0.037). However, one factor contained only two items and the scree-plot suggested a one-factor solution would be a better fit (see Additional file 2: Figure S1). Confirmatory factor analysis in the other half of the sample considered this five-factor solution (RMSEA = 0.069; CFI = 0.931; TLI = 0.921), along with a four-factor solution identified in previous studies of the CSI [70] and Dutch CSI[71] (RMSEA = 0.073; CFI = 0.921; TLI = 0.912), and a bifactor solution proposed by Cuesta-Vargas et al. [82], comprising one general factor and four orthogonal factors (RMSEA = 0.063; CFI = 0.945; TLI = 0.934)—see Table 1 for factor loadings. Model fit was acceptable for all models but marginally better for the bifactor model. The bifactor model provides support for the use of the CSI as a total score since this model involves the presence of a general factor. This is further supported by the CSI items showing excellent internal consistency (Cronbach’s *α* = 0.907 with all inter-item correlations highly significant (*p* < 0.01)). Taken together these results indicate that the CSI total score provides a valid and reliable assessment of the central sensitisation in autistic adults.

*Multigroup exploratory factor analysis* Configural equivalence was supported by EFA indicating the same number of factors to extract in both genders, and further supported in the CFA sample where the bifactor model was observed to provide optimal fit in both genders. A metric equivalent model, fixing factor loadings to the same values across genders, fit the data significantly worse than a configural model, where loadings were allowed to differ (*χ*^2^(24) = 37.7, *p* = 0.038). A partial metric equivalence model, fixing factor loadings to the same values except for items 11 and 16, did not fit significantly worse (*χ*^2^(22) = 24.9, *p* = 0.303). A scalar equivalence model fixing thresholds to the same values did not fit worse than the partial-matric equivalence model (*χ*^2^(24) = 37.7, *p* = 0.038). Taken together this indicates that the CSI captures the same construct in both men and women but there is some indication of differential item functioning, which may limit the ability to compare scores across genders.

**Table 1:** Item content and factor loadings (*λ*) of the CSI items

CSI item		General factor	F1λ	F2λ	F3λ	F4λ
1	I feel unrefreshed when I wake up in the morning	.560	.659			
2	My muscles feel stiff and achy	.705	− .077			
3	I have anxiety attacks	.593		.189		
4	I grind or clench my teeth	.428			.396	
5	I have problems with diarrhoea and/or constipation	.538	− .018			
6	I need help in performing my daily activities	.566	− .110			
7	I am sensitive to bright lights	.638			.051	
8	I get tired very easily when I am physically active	.730	.041			
9	I feel pain all over my body	.821	− .120			
10	I have headaches	.504			.067	
11	I feel discomfort in my bladder and/or burning when I urinate	.522				1.756
12	I do not sleep well	.557	.413			
13	I have difficulty concentrating	.640		.628		
14	I have skin problems such as dryness, itchiness, or rashes	.515	.090			
15	Stress makes my physical symptoms get worse	.657		− .021		
16	I feel sad or depressed	.528		.274		
17	I have low energy	.780	.194			
18	I have muscle tension in my neck and shoulders	.681	.078			
19	I have pain in my jaw	.638			.790	
20	Certain smells, such as perfumes, make me feel dizzy and nauseated	.605			.077	
21	I have to urinate frequently	.450				.151
22	My legs feel uncomfortable and restless when I am trying to go to sleep at night	.543	− .011			
23	I have difficulty remembering things	.490		.432		
24	I suffered trauma as a child	.510		.173		
25	I have pain in my pelvic area	.661				.059

### Group differences

Assumptions of independence and normality were tested, where appropriate, with histograms and Shapiro–Wilk tests. Homogeneity was examined using Levene’s Tests. Group differences were calculated using independent *t*-tests. In the diagnosed CSS versus no CSS groups, CSI scores were significantly higher and physical health ratings significantly lower, as expected (Table 2). The diagnosed CSS group also reported significantly lower SPQ scores, indicating greater sensory sensitivity. Participants with a CSS reported significantly more anxiety and depression, and lower subjective wellbeing, than those without a CSS. There was no significant group difference in autistic traits. As some measures were completed in different waves of the NAR and/or some participants had not completed them, the sample sizes differed across measures.

**Table 2:** Group differences between low and high CSI, diagnosed CSS and no diagnosis, men and women

Measure		Group					
		No CSS	Diagnosed CSS	Low CSI	High CSI	Men	Women
CSI	*N*	765	208	391	582	410	563
	Mean (SD)	40.4 (14.65)	55.3 (13.41)	28.4 (8.46)	53.8 (10.04)	37.1 (14.94)	48.3 (14.39)
	*T* Test	*t* (971) = − 13.214 *p* < .001**		*t* (971) = − 41.164 *p* < .001**		*t* (971) = − 11.774 *p* < .001**	
	*d*, 95% CI [LL UL]	1.06***, [− 17.08 − 12.67]		2.74***, [− 26.61 − 24.19]		0.76**, [− 13.04 − 9.32]	
AQ-short	*N*	765	208	391	582	410	563
	Mean (SD)	83.3 (10.92)	85.0 (10.69)	81.2 (11.15)	85.3 (10.40)	83.4 (11.61)	83.8 (10.34)
	*T* Test	*t* (971) = − 2.113 *p* = .035		*t* (971) = − 5.830 *p* < .001**		*t* (971) = − .499 *p* = .618	
	*d*, 95% CI [LL UL]	*N/A,* [− 3.46 − 0.13]		0.38*, [− 5.46 − 2.71]		*N/A*, [− 1.74 1.04]	
SPQ	*N*	313	80	173	220	186	207
	Mean (SD)	45.9 (15.00)	38.3 (13.80)	50.5 (14.89)	39.5 (13.40)	47.1 (15.50)	41.8 (14.24)
	*T* Test	*t* (391) = 4.133 *p* < .001**		*t* (391) = 7.628 *p* < .001**		*t* (391) = 3.539 *p* < .001**	
	*d*, 95% CI [LL UL]	0.53**, [4.01 11.28]		0.77**, [8.10 13.72]		0.36*, [2.36 8.26]	
HADS-A	*N*	507	129	276	360	276	360
	Mean (SD)	9.2 (4.37)	11.7 (4.12)	7.3 (3.89)	11.5 (3.92)	8.5 (4.55)	10.5 (4.14)
	*T* Test	*t* (634) = − 5.832 *p* < .001**		*t* (634) = − 13.584 *p* < .001**		*t* (634) = − 5.809 *p* < .001**	
	*d*, 95% CI [LL UL]	0.58**, [− 3.32 − 1.65]		1.09***, [− 4.86 − 3.63]		0.46**, [− 2.69 − 1.33]	
HADS-D	*N*	507	129	276	360	276	360
	Mean (SD)	7.0 (4.83)	9.2 (4.79)	5.5 (4.28)	9.0 (4.82)	7.1 (4.99)	7.7 (4.83)
	*T* Test	*t* (634) = − 4.734 *p* < .001**		*t* (634) = − 9.411 *p* < .001**		*t* (634) = − 1.597 *p* = .111	
	*d*, 95% CI [LL UL]	0.47**, [− 3.19 − 1.32]		0.76**, [− 4.18 − 2.74]		*N/A*, [− 1.40 0.14]	
Physical Health	*N*	765	208	391	582	410	563
	Mean (SD)	6.6 (1.47)	5.2 (1.52)	7.1 (1.21)	5.8 (1.57)	6.6 (1.55)	6.1 (1.59)
	*T* Test	*t* (971) = 12.121 *p* < .001**		*t* (971) = 14.486 *p* < .001**		*t* (971) = 4.228 *p* < .001**	
	*d*, 95% CI [LL UL]	0.94***, [1.17 1.63]		0.97***, [1.18 1.55]		0.28*, [0.23 0.63]	
Subjective Wellbeing	*N*	336	82	185	233	199	219
	Mean (SD)	29.9 (8.03)	25.6 (7.39)	32.6 (7.33)	26.2 (7.53)	30.4 (8.53)	27.8 (7.45)
	*T* Test	*t* (416) = 4.362 *p* < .001**		*t* (416) = 8.655 *p* < .001**		*t* (416) = 3.289 *p* = .001*	
	*d*, 95% CI [LL UL]	0.55**, [2.33 6.16]		0.85***, [4.90 7.78]		0.32*, [1.03 4.11]	

Bonferroni corrected *p* value = .002. *p < .002; **p < .001. Effect size *d* *small effect; **medium; ***large

CSI, Central Sensitization Inventory; SPQ, Sensory Perception Quotient; AQ-Short, Autism Quotient-Short; HADS-A, Hospital Anxiety and Depression Scale-Anxiety; HADS-D, Hospital Anxiety and Depression Scale-Depression

Gender differences were significant for CSI, physical health, SPQ, anxiety and subjective wellbeing, with women obtaining more severe scores for each of these instruments. Chi square tests of independence were used to analyse the relationship between gender, CSS diagnosis, and CSI score group (see Table 2). Women were significantly over-represented in both the diagnosed CSS group, *X*^2^ (2, *N* = 973) = 33.68, *p* < 0.001 and the High CSI group, *X*^2^ (2, *N* = 973) = 89.0, *p* < 0.001.

### Exploratory analyses

The following analyses were undertaken on an exploratory basis, to provide future research directions.

#### Hierarchical regression and path analysis

The data was tested to ensure relevant assumptions were met. Our sample size was considered adequate. Some predictor variables were correlated with each other (see Table 3) however multicollinearity tests indicated that this was not a concern for the regression analysis, with tolerance ranging from 0.832 (anxiety) to 0.919 (age) and VIFs ranging from 1.088 (age) to 1.201 (anxiety). The data also met the assumption of independent errors (Durbin-Watson value = 1.832). Histograms and scatterplots were utilised to ascertain that the assumptions of normality, heteroscedasticity and linearity were met.

**Table 3:** Correlation matrix for regression (*n* = 359)

	CSI	Gender	Age	AQ	Anxiety	SPQ
CSI	1.00	.349^**^	0.05	.250^**^	.629^**^	− .460^**^
Gender	.349^**^	1.00	− .261^**^	0.02	.246^**^	− .185^**^
Age	0.05	− .261^**^	1.00	0.09	0.00	0.04
AQ	.250^**^	0.02	0.09	1.00	.243^**^	− .250^**^
Anxiety	.629^**^	.246^**^	0.00	.243^**^	1.00	− .311^**^
SPQ	− .460^**^	− .185^**^	0.04	− .250^**^	− .311^**^	1.00

*p* value = .05; *p < .01; ** *p* < .001**

The relationships between the main variables were analysed using a four-stage hierarchical multiple regression with CSI score as the dependent variable. Stage one included age and gender, stage two added autistic traits (AQ), stage three sensory sensitivity (SPQ) and stage four anxiety (HADS-A) (Table 4).

**Table 4:** Hierarchical regression analysis (*n* = 359)

	Stage one			Stage two			Stage three		
	Beta	*t*	*p*	Beta	*t*	*p*	Beta	*t*	*p*
(Constant)		7.765	< .001***		0.090	0.928		3.723	< .001***
Gender	0.388	7.628	< .001***	0.377	7.633	< .001***	0.208	5.211	< .001***
Age	0.148	2.912	.004**	0.124	2.499	0.013*	0.106	2.747	0.006**
AQ score				0.231	4.819	< .001***	0.052	1.340	0.181
Anxiety							0.483	11.884	< .001***
SPQ score							− 0.263	− 6.565	< .001***

*R*^2^ = .142 for Model 1: Δ*R*^2^ = .053 for Model 2 (*p* < .001): Δ*R*^2^ = .320 for Model 3 (*p* < .001). *p < .05; **p < .01; ***p < .001

Stage one of the regression analysis showed that gender and age were both significantly associated with CSS symptoms and accounted for 14.2% of the variability in CSI scores. Stage two accounted for 18.8% of the variability in CSI scores and contributed significantly to the regression model, *F* (3,355) = 28.67, *p* < 0.001, with AQ scores explaining 5.3% additional variance in CSI scores. Sensory sensitivity contributed to a further 12.6% of the variance (*F* (4,354) = 41.92, *p* < 0.001) and anxiety to 19.4% of the variance (*F* (5,353) = 75.09, *p* < 0.001). In total the model accounted for 50.9% of the variance in CSI scores. Level of autism traits (AQ) was not a significant predictor when anxiety and sensory sensitivity were included.

**Fig. 1:**
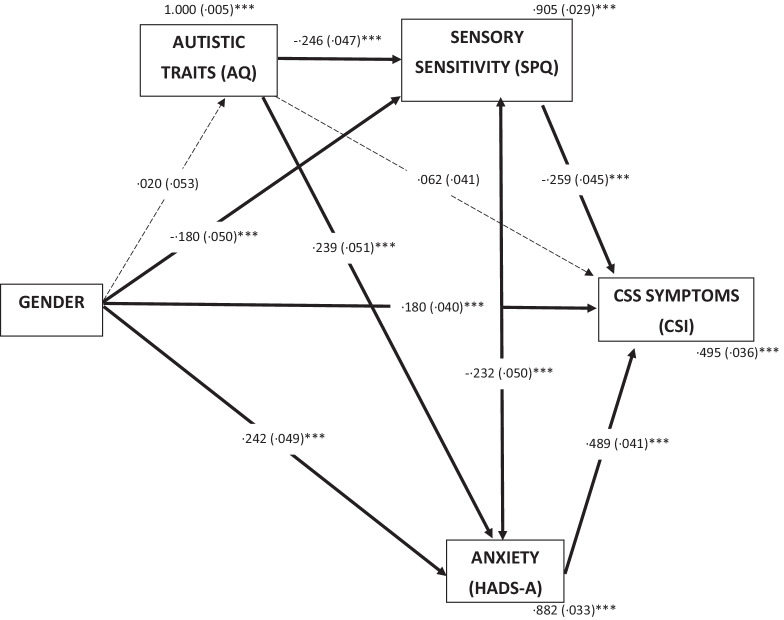
Mediation model of CSS symptoms in autistic adults. Standardized path coefficients (S.E), covariates and residual variance shown. *p < .05; **p < .01; ***p < .001

To explore these associations further two path models were performed. Path model one included gender as a predictor variable, sensory sensitivity (SPQ), autistic traits (AQ) and anxiety (HADS-A) as mediator variables, and CSS symptoms (CSI) as an outcome variable (see Fig. 1). The rationale for this approach was that gender is usually established earlier in life, whereas CSS symptoms are more likely to manifest later on. Likewise, autistic traits and sensory sensitivity are both usually present from infancy, and therefore were presented earlier in the model. In this analysis, gender significantly predicted sensory sensitivity (*p* < 0.001), anxiety (*p* < 0.001), and CSS symptoms (*p* < 0.001). Autistic traits significantly predicted both sensory sensitivity (*p* < 0.001) and anxiety (*p* < 0.001). Sensory sensitivity significantly predicted CSI scores (*p* < 0.001) as did anxiety (*p* < 0.001), but autistic traits did not (*p* = 0.127). Anxiety, sensory sensitivity, and gender together accounted for 50.5% of the variance in CSI scores, *R*^2^ = 0.505. The only indirect effect that was non-significant was that of gender on CSS symptoms via autistic traits.

**Fig. 2:**
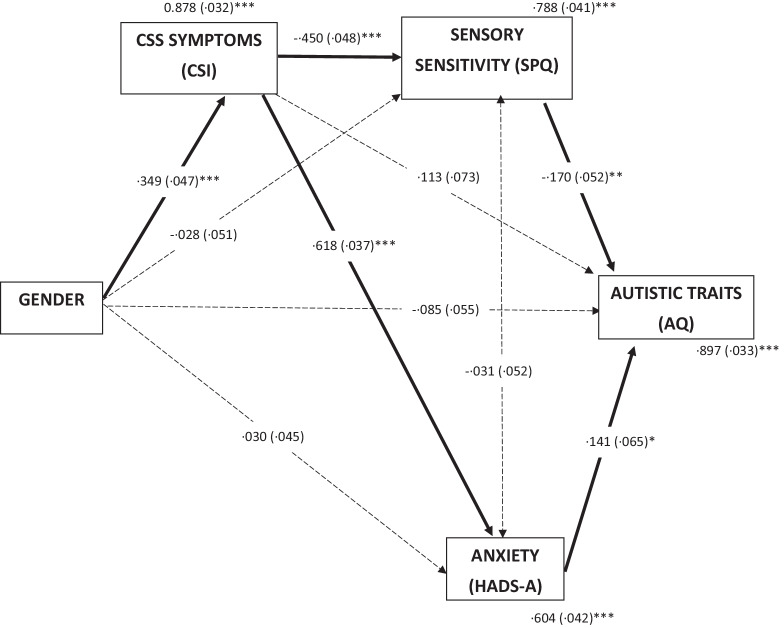
Alternative mediation model of CSS symptoms in autistic adults. Standardized path coefficients (S.E), covariates and residual variance shown. *p < .05; **p < .01; ***p < .001

Path model two was performed in which the position of autistic traits and CSS symptoms were reversed, i.e. with gender as a predictor variable, sensory sensitivity (SPQ), CSS symptoms (CSI) and anxiety (HADS-A) as mediator variables, and autistic traits (AQ) as an outcome variable (see Fig. 2). This was based on helpful recommendations from reviewers, given the issues with interpretation of path-analyses in cross-sectional data. In this analysis, gender significantly predicted CSS symptoms (*p* < 0.001). CSS symptoms significantly predicted both sensory sensitivity (*p* < 0.001) and anxiety (*p* < 0.001). Sensory sensitivity significantly predicted autistic traits (*p* < 0.01) as did anxiety (*p* < 0.05), but CSS symptoms did not (*p* = 0.120). Anxiety, sensory sensitivity, and gender together accounted for 10.3% of the variance in autistic traits, *R*^2^ = 0.103. There were no significant indirect effects. Together these two models indicated that differences in CSS symptoms between men and women are likely explained by differences in sensory sensitivity and anxiety, rather than by degree of autistic traits; whereas differences in degree of autistic traits are not directly related to gender.

## Discussion

This is the first study, to our knowledge, that directly considers an association between autism, central sensitisation, and CSS. In our large sample of autistic adults, 21% reported a CSS diagnosis of FMS, ME/CFS, IBS, RLS or TMJD and 60% scored at or above the clinical cut-off for a CSS on the CSI, suggesting that CSS symptoms are very common in autistic people.

A factor analysis of the CSI [70] was undertaken to test the measure’s construct validity in an autistic sample, as previous CSI studies have focussed on chronic pain and control groups [67, 71, 82]. The results supported the bi-factor model [82], and a highly internally consistent scale. Partial-metric equivalence was observed between genders with items 11 “I feel discomfort in my bladder and/or burning when I urinate” and 16 “I feel sad or depressed” identified as functioning differently between men and women. Given that the difference in loadings were relatively small (< 0.2), and that two out of 25 items were affected, the impact on the comparability of scores across males and females is likely to be small. It is unclear whether this observation is specific to autistic people or generalises to the population as a whole.

In our sample, the mean CSI score for those with a diagnosed CSS was 55.3, slightly higher than the mean score of 52.4 Neblett et al. [67] found in their study of CSS patients establishing the clinical cut-off of 40 on the CSI. However, the mean CSI score for autistic participants without a diagnosed CSS was 40.6; a score far higher than that of Neblett’s control group (30.9) and closer instead to the mean score of 40.9 in Neblett’s non-CSS chronic pain patients. This suggests that CSS symptoms such as pain and fatigue are very common in autistic individuals and possibly more prevalent than in the general population.

There are many theoretical reasons why autism and CSS might be linked with each other. Sensory processing differences are a core feature of autism [13] with autistic people reporting greater sensory sensitivity [15] than the general population. People with CSS also experience sensory sensitivity [14] but in this case it is more clearly associated with central sensitisation [5]. Our results demonstrated that autistic people with greater sensory sensitivity also had more CSS symptoms. Whilst it is possible that this is due to an overlap of symptoms or traits between the CSI and the autistic experience, this also fits with recent research indicating that generalised or multi-sensory sensitivity may be a risk factor for developing central sensitisation or chronic pain [86–89].

Our results also showed that, as predicted, higher scores on the CSI were associated with greater sensory sensitivity, greater anxiety and lower subjective well-being. Whilst higher scores on the CSI also appeared to be associated with higher autistic traits, the path analysis conducted suggested that sensory sensitivity and anxiety mediated this relationship. This suggests that sensory sensitivity and anxiety may increase vulnerability to CSS, rather than autism per se.

The finding that anxiety acted as a mediator in this model is in line with previous research on pain in autism that identified anxiety as a contributor to pain levels [29, 69]. CSS research also suggests that anxiety may contribute to both the development of central sensitisation and the severity of symptoms [90, 91]. Although we only considered anxiety in our exploratory analyses, existing research suggests that many mental health conditions could affect the incidence of CSS symptoms in autistic people; high anxiety [33], chronic stress [92] and PTSD [33, 93] have all been associated with CSS, and are also more common in the autistic community [29, 31, 34, 94] than the general population. Other psychological factors could also be important; for example, some research suggests chronic illness severity might be affected by illness beliefs and coping mechanisms [95] and how these relate to autism has not been explored.

Just as previously reported in the general population [57, 67], there were clear gender differences in this study, with women over-represented for both CSS diagnoses and number and severity of CSS symptoms. Women also showed greater sensory sensitivity and reported greater anxiety, depression and lower subjective wellbeing. Previous research into sensory sensitivity in autism has been mixed when considering gender differences [15, 96]. Recent studies on the SPQ, both on data within the Netherlands Autism Register (of which this dataset is also a subsample) and outside, found that autistic women had higher sensory sensitivity than both autistic men [72] and non-autistic women [24]. Research within the general population also suggests that women may be more sensitive than men across a range of modalities [58, 97], with hormones thought to play a key role [98]. However consideration needs to be given to issues like gender bias [63]. The results of this study suggest that autistic women might be more likely to experience central sensitisation and related CSS than autistic men.

Whilst not a focus of our study, it is also possible that our results could be explained by neuroimmune and genetic differences in the autistic population. Recent research into joint hypermobility, hypermobility spectrum disorders and the Ehlers-Danlos syndromes (EDS) [52, 56, 99–102] as well as mast cell activation syndrome (MCAS) [103, 104] and dysautonomia [105, 106] has suggested that autistic people, and people with ADHD, are over-represented in these conditions. HSD and EDS are also associated with chronic pain, and are often found to co-occur with, or underly, CSS diagnoses [56]. We did not include questions on joint hypermobility in this study, but future research might consider including these conditions.

Whilst our results show quite clearly that autistic people experience a lot of symptoms associated with central sensitisation, how these symptoms translate to CSS diagnoses is more difficult to establish. Firstly, not all conditions considered to be a CSS were included in our analysis; for example, migraine was not included in our list of CSS, and only 733 of the 973 participants that completed CSI Part A also completed the co-occurring conditions section. This means that the number of participants with a diagnosed CSS is likely to be understated in the current sample. We also did not have CSS diagnosis dates available in our data so cannot examine whether autistic people with CSS were diagnosed with CSS before or after their autism diagnosis. This is important because there are many nuanced difficulties that may exist around diagnosis. For example, autistic people are more likely to experience difficulties accessing healthcare [42, 107], communicating with clinicians [108, 109], and also express pain differently to the general population [29, 110]. There is also a danger of diagnostic overshadowing, where symptoms of CSS may be incorrectly attributed to an existing autism diagnosis; research shows that clinicians are often uninformed about co-occurring conditions in autism [111]. In addition, fatigue may be attributed to autistic masking [112] or burnout [113] by health professionals or the autistic person themselves, when actually it is a sign of an underlying CSS.

Autism diagnostic issues might also explain why an association between autism and central sensitisation has thus far been largely overlooked. Historically, autism has been classified as a social and communication disorder [114], with sensory issues only included in the most recent DSM criteria [115]. An autism diagnosis is still predominantly based on behaviour in childhood, with a considerable gender bias such that women tend to be underdiagnosed [64] or diagnosed later [65]. Increased understanding of the lived experience of autism has improved awareness of the many co-occurring health issues autistic people experience [116] but this is not reflected in the current diagnostic criteria [115, 117] or in measures aiming to assess autistic traits, such as the AQ [74], where large dimensions of the autistic experience are excluded [118]. It could be the case that the CSI has captured physical features that have always been common in the autistic population, but not recognised because they were not obvious to the external observer.

Clinically, this study has important implications. We found that the relationship between autistic traits and CSS symptoms was fully mediated by anxiety and sensory sensitivity. Autistic people often struggle to access mental health support or occupational therapy, particularly in adulthood [119, 120]. Our research suggests that increased anxiety and sensory sensitivity could have wider physical health implications, and longitudinal research could explore further whether interventions focussed on these aspects might mitigate the risk of autistic people developing a CSS later in life.

### Limitations

A strength of this study is that these data were reported as part of an ongoing data collection in the NAR volunteer register, with participants not explicitly primed as to the aims of the CSI data collection. Therefore, it is unlikely that these findings are inflated due to selection or attrition bias.

In terms of limitations, firstly, although we were able to include a large sample of autistic participants, we did not have a control or CSS only group. Future research including these groups would add additional power and allow for a greater exploration of the relationships between the main variables in the wider population. Our sample was also predominantly Dutch, with very few reporting to be a member of an ethnic minority. Future studies should try to recruit a more ethnically diverse sample.

Secondly, the variable ‘gender’ was assessed by asking participants to check one of three boxes (man/woman/other). Participants could have interpreted this as either ‘sex assigned at birth’ or as ‘gender of identification’. In many cases sex and gender will overlap but we know this will not apply to everyone. Participants indicating their gender as “other” were excluded due to the small sample size and this field was referred to throughout the study as “gender”. Since central sensitisation seems to be linked to sex and hormones [57, 98] in the research literature, and that autistic individuals are less likely to identify with their assigned sex at birth [121–123], it is important that further research is undertaken in which assigned sex at birth, gender, and hormonal influences are clearly delineated.

Another important limitation involves the wording of the CSI [70]. It is possible that the association between autism and CSS identified in this study relates more to an overlap of traits (particularly sensory sensitivity) than a true co-occurrence. It is also possible that central sensitisation is directly related to sensory sensitivity [86]. Furthermore, whilst the AQ is a widely used and reliable measure [68], its ability to capture the essence of what autism is, and measure this quantitatively, is limited [118, 124]. Further studies could utilise different illness-specific instruments and alternative instruments to the CSI and AQ to establish whether a relationship between autistic traits and CSS can be identified in different conditions.

There are many other aspects that may influence a possible association between autism and CSS that we were unable to measure in this study. For example, the SPQ [15] does not measure interoception, but research indicates interoceptive awareness is altered in autistic people [125] and also people with chronic pain [126]. Similarly, we did not include an alexithymia measure in our analyses, however alexithymia has been indicated in chronic pain [127], and is very common in autistic people [128]. Future research on autism and CSS would need to consider these phenomena.

In this study, CSS diagnoses were self-reported and not independently verified. It is possible that participants may have indicated conditions that were not formally clinically diagnosed. Diagnosis of CSS can be particularly difficult, with clinician bias, misdiagnosis and diagnostic inconsistency a continuing problem [129, 130]. Future studies could recruit via health services or incorporate additional questions to confirm CSS diagnoses. It is also possible that CSS diagnoses were underestimated in this study, partly due to not all participants completing sections on co-occurring conditions, and partly due to not including the ‘migraine’ diagnosis as this field also included ‘headache’ (see “Measures” for more details). It is also important to note that the concept of central sensitisation as a unifying underlying feature of CSS, often also referred to as “chronic overlapping pain conditions” (COPCs), is still relatively new, and research is still evolving in terms of which health conditions are considered to come under this umbrella [46, 131], therefore conditions that were included as a CSS in this study may change category in the future. Finally, as a cross-sectional study, this research is limited when exploring cause and effect, such as through the regression and path analyses, and any inferences drawn need to be treated with caution. Longitudinal studies may be able to shed more light on how and why autism and CSS might be related.

## Conclusions

In conclusion, in a large sample of autistic adults, 21% had a diagnosis of at least one CSS included in this study, and the majority experienced symptoms of central sensitisation, with 60% scoring at or above the clinical cut-off on a widely used screening measure.

The results suggest that clinicians need to be aware of a possible association between CSS and autism and mindful of the potential risk of misdiagnosis or diagnostic overshadowing. This is particularly true for women, in whom autism is underdiagnosed [64]. Future research could consider whether autism screening in the diagnostic CSS diagnostic process might be appropriate, for example, and consider whether physical symptoms in autism may warrant evaluation for a CSS bearing in mind the high percentage of autistic adults in this sample that experienced CSS symptoms but did not have a CSS diagnosis.

Most importantly, practitioners should recognise that physical health symptoms and co-occurring conditions are common in autistic people, and that these symptoms can be treated to improve overall quality of life.

## Supplementary Information

**Additional file 1: Table S1**. Number of participants per diagnosis (41 participants had more than one diagnosis). **Table S2**. Fit statistics for exploratory factor analysis (Promax rotation). **Table S3**. Fit statistics for exploratory factor analysis (bi-geomin rotation ML estimator). **Table S4**. Fit statistics for confirmatory factor analysis using robust weighted least squares estimator (WLSMV).

**Additional file 2: Figure S1**. Scree plot for exploratory factor analysis.

## Data Availability

The datasets generated and/or analysed during the current study are available in the Netherlands Autism Register, www.nederland-sautismeregister.nl/english/.

## Abbreviations

AQ: Autism Spectrum Quotient
CSI: Central Sensitization Inventory
CSS: Central Sensitivity Syndromes
FMS: Fibromyalgia Syndrome
HADS: Hospital Anxiety and Depression Scale
IBS: Irritable Bowel Syndrome
ME/CFS: Myalgic Encephalomyelitis/Chronic Fatigue Syndrome
NAR: Netherlands Autism Register
PTSD: Post-Traumatic Stress Disorder
SPQ: Sensory Perception Quotient
TMJD: Temperomandibular Joint Disorder
