# Supplementary material for: Autism and chronic ill health: an observational study of symptoms and diagnoses of central sensitivity syndromes in autistic adults
Source: Mol Autism. 2022 Feb 14;13:7. doi: 10.1186/s13229-022-00486-6 (PMC8842858; doi:10.1186/s13229-022-00486-6)
Supplement: Supplementary file 1 — Additional file 1: Table S1. Number of participants per diagnosis (41 participants had more than one diagnosis). Table S2. Fit statistics for exploratory factor analysis (Promax rotation). Table S3. Fit statistics for exploratory factor analysis (bi-geomin rotation ML estimator). Table S4. Fit statistics for confirmatory factor analysis using robust weighted least squares estimator (WLSMV). [file 13229_2022_486_MOESM1_ESM.docx]

**Additional file**

**Additional file 1: Table S1. Number of participants per diagnosis (41 participants had more than one diagnosis)**

|  | Men | Women | Total |
| --- | --- | --- | --- |
| Fibromyalgia | 3 | 16 | 19 |
| Chronic Fatigue Syndrome | 9 | 24 | 33 |
| Restless Legs Syndrome | 16 | 35 | 51 |
| Irritable Bowel Syndrome | 31 | 113 | 144 |
| Temporomandibular Joint Dysfunction | 0 | 8 | 8 |

**Additional file 1: Table S2. Fit statistics for exploratory factor analysis (Promax rotation)**

|  | **CHI^2^** | | |  |  |
| --- | --- | --- | --- | --- | --- |
| **MODEL** | **VALUE** | **DF** | **P** | **RMSEA** | **SRMR** |
| One factor | 1267.567 | 275 | <.001 | 0.084 | 0.073 |
| Two factor | 863.717 | 251 | <.001 | 0.069 | 0.058 |
| Three factor | 699.481 | 228 | <.001 | 0.063 | 0.051 |
| Four factor | 556.627 | 206 | <.001 | 0.058 | 0.045 |
| Five factor | 432.832 | 185 | <.001 | 0.051 | 0.037 |

**Additional file 1: Table S3. Fit statistics for exploratory factor analysis (bi-geomin rotation ML estimator)**

|  | **CHI^2^** | | |  |  |  |  |
| --- | --- | --- | --- | --- | --- | --- | --- |
| **MODEL** | **VALUE** | **DF** | **P** | **RMSEA** | **CFI** | **TLI** | **SRMR** |
| Two factor | 863.717 | 251 | <.001 | 0.069 | 0.922 | 0.907 | 0.058 |
| Three factor | 699.481 | 228 | <.001 | 0.063 | 0.94 | 0.921 | 0.051 |
| Four factor | 556.627 | 206 | <.001 | 0.058 | 0.956 | 0.935 | 0.045 |
| Five factor | 432.832 | 185 | <.001 | 0.051 | 0.969 | 0.949 | 0.037 |
| Six factor | 328.072 | 165 | <.001 | 0.044 | 0.979 | 0.962 | 0.031 |

**Additional file 1: Table S4. Fit statistics for confirmatory factor analysis using robust weighted least squares estimator (WLSMV)**

|  | **WLSMV ESTIMATOR** | | | | | | |
| --- | --- | --- | --- | --- | --- | --- | --- |
| **MODEL** | **CHI^2^** | **DF** | **P** | **RMSEA** | **CFI** | **TLI** | **SRMR** |
| One factor | 1166.582 | 275 | <.001 | 0.079 | 0.906 | 0.898 | 0.056 |
| Two factor | 1040.915 | 274 | <.001 | 0.074 | 0.919 | 0.912 | 0.053 |
| Three factor | 955.719 | 272 | <.001 | 0.07 | 0.928 | 0.921 | 0.051 |
| Four factor, Mayer model | 1015.177 | 269 | <.001 | 0.073 | 0.921 | 0.912 | 0.052 |
| **Bifactor, Cuesta-Vargas model** | **769.549** | **250** | **<.001** | **0.063** | **0.945** | **0.934** | **0.046** |
| Five Factor model | 923.445 | 265 | <.001 | 0.069 | 0.931 | 0.921 | 0.049 |
