# Supplementary figures and images for: Autism and chronic ill health: an observational study of symptoms and diagnoses of central sensitivity syndromes in autistic adults
Source: Mol Autism. 2022 Feb 14;13:7. doi: 10.1186/s13229-022-00486-6 (PMC8842858; doi:10.1186/s13229-022-00486-6)

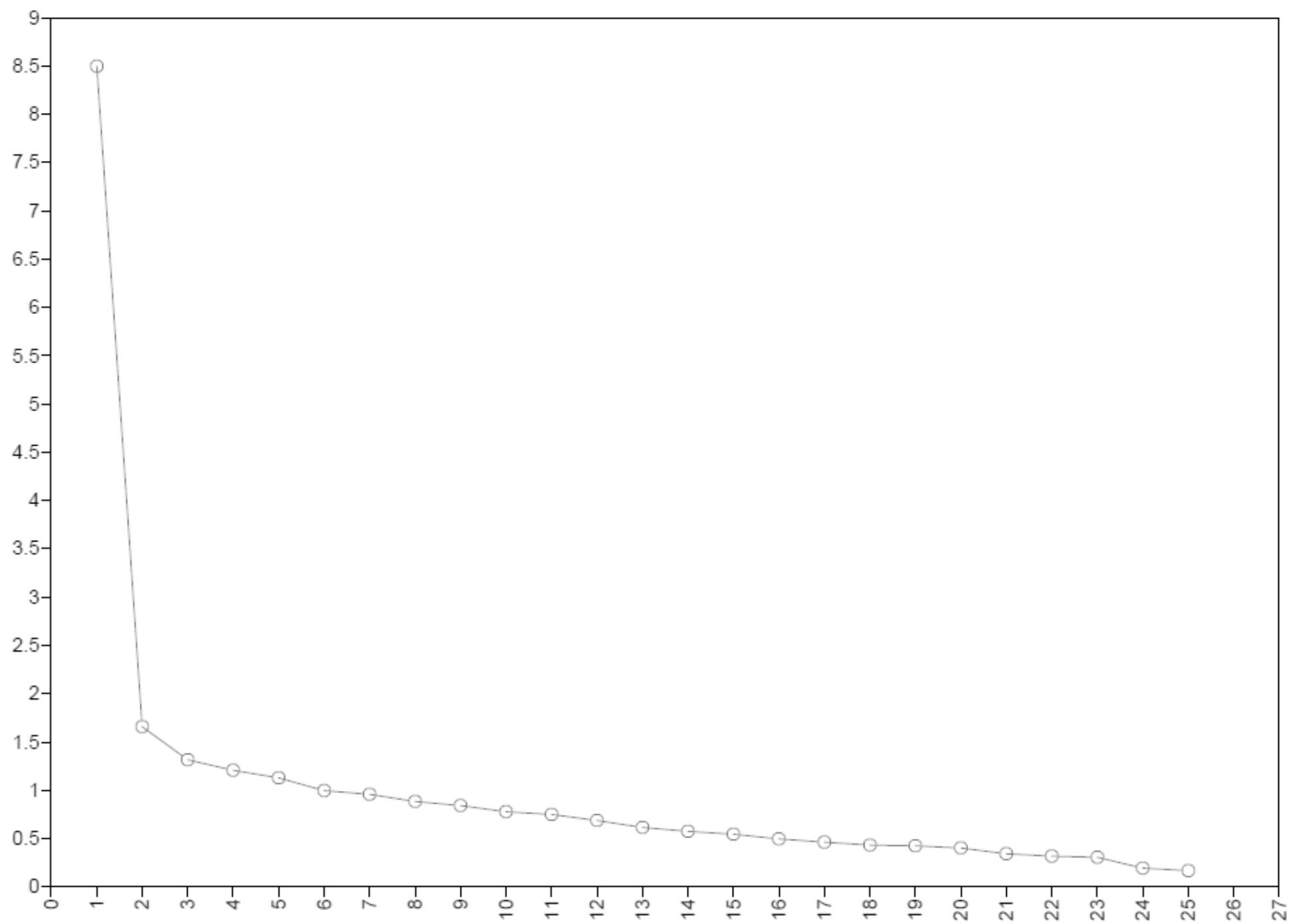

Supplement: Supplementary file 2 — Additional file 2: Figure S1. Scree plot for exploratory factor analysis. [file 13229_2022_486_MOESM2_ESM.pdf]
